# Central venous catheter-related bloodstream infection - prevention, records and auditing

**DOI:** 10.1186/2197-425X-3-S1-A887

**Published:** 2015-10-01

**Authors:** AV Varela, P Vala, M Dykyy, R Almeida, J Arduán, A Gonçalves, MA Pereira, I Hubert, C Granja

**Affiliations:** Centro Hospitalar do Algarve, Intensive Care Unit 1-Faro, Hospital de Faro, Faro, Portugal; Centro Hospitalar do Algarve, Hospital de Faro, Faro, Portugal

## Introduction

Central venous catheterization is commonly used in critically ill patients and may cause different complications, including infection. This type of infection contributes to morbidity, mortality and, as a consequence, to the increase in health care costs.

## Objectives

Evaluation of the incidence and adherence of the ICU team to an implemented protocol for prevention of central venous catheter- related bloodstream infection (CVCRBI).

## Methods

Exploratory-descriptive research based in auditing an implemented protocol through the use of checklists. After CVC- related bloodstream infection prevention protocol implementation, auditing was made during the period of 26 of November of 2014 to 3 of March of 2015. Population study: 17 doctors and 36 nurses were under observation. Auditing was made in random labor time of the investigators. Auditing checklists:

1- evaluation during insertion of CVC;

2- evaluation during maintenance and removing of CVC.

## Results

From all the 185 in-patients in the ICU, 141 had CVC. Mean age was 61 years old, 54% were males, mean length of ICU stay was 7 days. Type of admission: 49% medical; 18% elective surgery; 23% urgent surgery; 7% trauma; 3% potential organ donors. Mean SAPS II was 48; mean SOFA was 7. Overall catheter day's was 1196 that corresponds to 1,7% of CVCRBI. A total of 119 auditings were made (23 during insertion; 89 during maintenance and 3 during removal).

Audit results: compliance with protocol during insertion of CVC - 87%; compliance with protocol during maintenance was 83%.

## Conclusions

During the audit period we had a low incidence of CVCRBI. Compliance with implemented protocol was very high. During the same period the ICU mortality rate decreased. Although direct correlation can't be made, we might speculate, that the incidence of CVCRBI's could aid, in same way, to this decrease in mortality.

